# TSPO protein binding partners in bacteria, animals, and plants

**DOI:** 10.1007/s10863-021-09905-4

**Published:** 2021-06-30

**Authors:** Carrie Hiser, Beronda L. Montgomery, Shelagh Ferguson-Miller

**Affiliations:** 1 Department of Biochemistry and Molecular Biology, Michigan State University, East Lansing, MI 48824 USA; 2 Department of Energy Plant Research Laboratory, Michigan State University, East Lansing, MI 48824 USA; 3 Department of Microbiology and Molecular Genetics, Michigan State University, East Lansing, MI 48824 USA

**Keywords:** TSPO, Protein–protein interactions, Autophagy, VDAC, NADPH oxidase, 14-3-3 proteins

## Abstract

The ancient membrane protein TSPO is phylogenetically widespread from archaea and bacteria to insects, vertebrates, plants, and fungi. TSPO’s primary amino acid sequence is only modestly conserved between diverse species, although its five transmembrane helical structure appears mainly conserved. Its cellular location and orientation in membranes have been reported to vary between species and tissues, with implications for potential diverse binding partners and function. Most TSPO functions relate to stress-induced changes in metabolism, but in many cases it is unclear how TSPO itself functions—whether as a receptor, a sensor, a transporter, or a translocator. Much evidence suggests that TSPO acts indirectly by association with various protein binding partners or with endogenous or exogenous ligands. In this review, we focus on proteins that have most commonly been invoked as TSPO binding partners. We suggest that TSPO was originally a bacterial receptor/stress sensor associated with porphyrin binding as its most ancestral function and that it later developed additional stress-related roles in eukaryotes as its ability to bind new partners evolved.

## Introduction

TSPO was first discovered as a receptor for benzodiazepine drugs in mammalian peripheral tissues, distinct from the GABA receptor of the central nervous system that also interacts with benzodiazepine drugs (Braestrup et al. 1977; Braestrup and Squires 1977). TSPO was therefore named the peripheral benzodiazepine receptor (PBR). Independently discovered in bacteria and named the tryptophan-rich sensory protein (TspO) (Yeliseev and Kaplan 1995), it was also called the translocator protein 18kD (Papadopoulos et al. 2006) to reflect both its high tryptophan content and the evolving view that it could be a sensor or translocator rather than solely a receptor for endogenous benzodiazepine-like molecules. In mammals, TSPO is expressed in most, if not all, tissues (Verma and Snyder 1989; Papadopoulos et al. 2006; Batarseh and Papadopoulos 2010; Fan et al. 2012; Gatliff et al. 2014) but is particularly enriched in steroidogenic tissues (Verma and Snyder 1989; Gavish et al. 1992; Papadopoulos et al. 1997; Lacapere and Papadopoulos 2003; Tu et al. 2014) and secretory and glandular tissues (Batarseh et al. 2010). However, this membrane protein is phylogenetically widespread from archaea and bacteria to insects, vertebrates, plants, and fungi (Lindemann et al. 2004; Chapalain et al. 2009; Fan et al. 2012), yet it is not ubiquitous. TSPO genes are notably absent in two prokaryotes widely used as laboratory organisms—*E. coli* and *Saccharomyces cerevisiae* (Fan et al. 2012).

## TSPO structure and location

### Conservation of sequence and structure

**Fig. 1:**
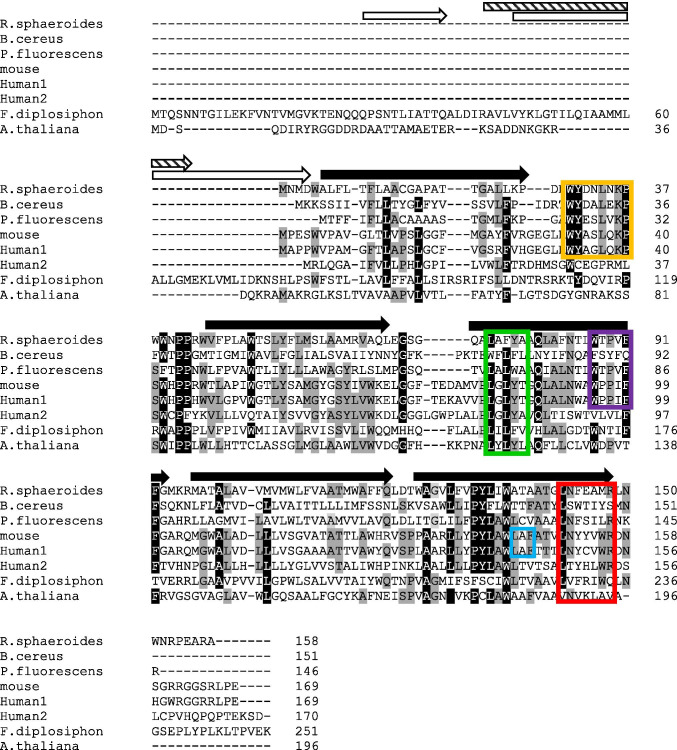
Sequence alignment of bacterial, animal, and plant TSPO proteins. Alignment was made with Clustal Omega (Madeira et al. 2019). Black arrows indicate crystallographically-defined transmembrane helices in *Rhodobacter sphaeroides* TSPO (Li et al. 2016). White arrows indicate additional predicted N-terminal helices of *Arabidopsis thaliana* TSPO (Jurkiewicz et al. 2020). The striped arrow indicates an additional predicted N-terminal helix in *Fremyella diplosiphon* TSPO1 (Busch et al. 2017). The LAF and CRAC motifs are outlined in blue and red, respectively. The AIM motif (Hachez et al. 2014) is outlined in green, the 14-3-3 binding motif (Aghazadeh et al. 2012) is outlined in yellow and the WxPxF motif (Li et al. 2016) is outlined in purple. Amino acids that are 80–100% identical are shown in black with white letters; those that are 50–70% identical are shown in gray

The widespread presence of TSPO in the biome correlates with its ancient origin (Zeng and Kaplan 2001; Fan et al. 2012; Leneveu-Jenvrin et al. 2014; Li et al. 2016; Veenman et al. 2016); thus, it is not surprising to find only modest amino acid sequence conservation between TSPOs from widely diverse species (Fig. 1). There is 91–95% identity between seven *Pseudomonas* strains (Leneveu-Jenvrin et al. 2014) and 60% identity between two *Rhodobacter* strains (Yeliseev and Kaplan 1995), but only 23% identity between the sequences of the unrelated bacteria *Rhodobacter sphaeroides* and *Bacillus cereus* (Li et al. 2016). Mammalian TSPO sequences average about 80% amino acid conservation amongst themselves (Chen and Guilarte 2008), but are generally only ~ 30–35% identical to bacterial TSPOs (Yeliseev and Kaplan 1995; Guo et al. 2015; Li et al. 2015c, 2016). TSPO amino acid sequence identity varies widely between plants and bacteria or mammals (12–36%) and even among plants themselves (38–85%) (Lindemann et al. 2004; Guillaumot et al. 2009a, b). An isoform, TSPO2, found in erythroid cells in mammals and birds (Fan et al. 2009; Nakazawa et al. 2009) is only about 35% identical to TSPO1 (Fan et al. 2009). Several additional isoforms have also been discovered in some cyanobacteria (Busch and Montgomery 2017; Busch et al. 2017) and plants (Frank et al. 2007; Fan et al. 2012) that vary in their degrees of similarity.

**Fig. 2:**
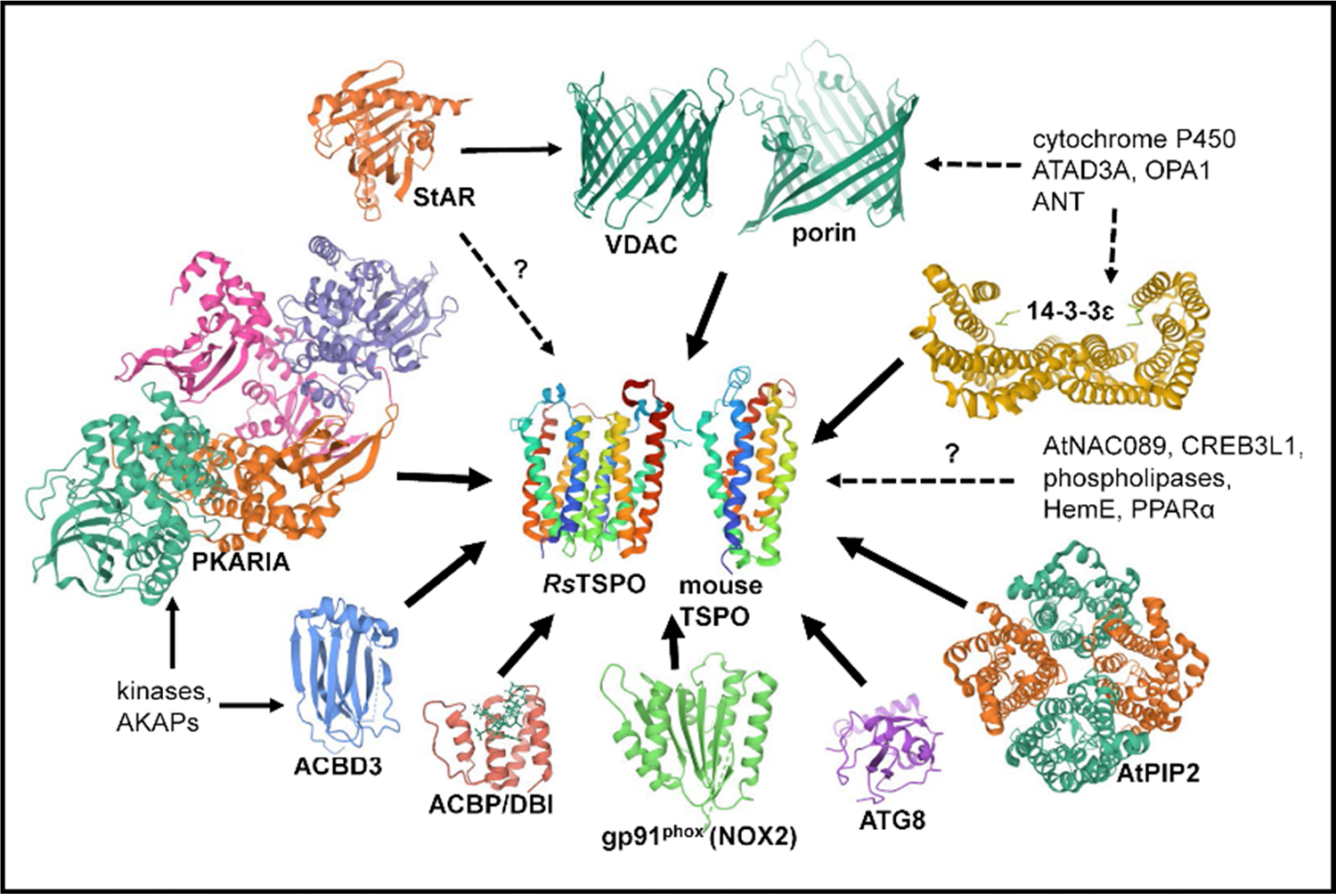
TSPO protein partners. The following protein structures were obtained from the RCSB PDB and images were created using Mol* (Sehnal et al. 2018) from the PBD website: *Rs*TSPO dimer (PDB:4UC1), mouse TSPO monomer (PDB:2NO2), human VDAC1 monomer (PDB:2JK4), *Rhodobacter capsulatus* porin trimer (PDB:2POR), human StAR-5 (PDB:2R55), human PKARIA (PDB:6NO7), human gp91^phox^ (PDB:3A1F), bovine ACBP (PDB:1ACA), human ACBD3 (PDB:5LZ1), human 14-3-3ɛ (PDB:2BR9), human 14-3-3ɣ (PDB:3UZD), and yeast ATG8 (PDB:2KWC). *Arabidopsis thaliana* PiP2;4 (PDB:6QIM) was included as there is no available structure for AtPIP2;7

The 3-dimensional structure of TSPO might be expected to be more highly conserved than the amino acid sequence, and indeed considerable similarity in overall fold is observed, suggesting an evolutionarily-conserved core structure of 5 transmembrane helices (Fig. 2, center). Yet some differences are observed between the available bacterial and mammalian structures. Crystal structures from two unrelated bacteria [*R. sphaeroides* (Li et al. 2015c) and *B. cereus* (Guo et al. 2015)] show the same 5 helical fold but the only published animal TSPO structures from mouse are NMR structures (Jaremko et al. 2014, 2015a, b) that differ in several regions compared to the bacterial structures. The researchers employing NMR attribute these differences to evolutionary distance between mammals and bacteria, crystal-packing artifacts in the bacterial x-ray structures, and the flexibility of a postulated intrinsically-disordered TSPO protein (Jaremko et al. 2015a, b). Other groups have concluded that the mouse protein may not be in its completely native conformation when analyzed by NMR due to destabilization by the detergents used in the protein purification, refolding, and NMR assay, that may result in some distortion (Li et al. 2015b, 2016; Zeng et al. 2018; Xia et al. 2019). A crystal structure of a mammalian TSPO will be needed to resolve this concern. So far, no structures of any plant or cyanobacterial TSPOs have been published, but given the conservation of certain amino acid sequences, especially highly-conserved tryptophans and histidines, it is likely that plant and cyanobacterial TSPOs also possess a similar core 5-helix structure. However, select TSPOs from *Arabidopsis thaliana* and some other plants (Lindemann et al. 2004; Guillaumot et al. 2009a; Jurkiewicz et al. 2020), as well as from the cyanobacterium *Fremyella diplosiphon* (Busch et al. 2017), have extended N-termini that may form additional alpha helices (Fig. 1).

The available bacterial structures show both monomer and dimer forms and the reported native oligomerization state of TSPO seems to vary between species and even between tissues in one organism. Indeed, multiple states have been found within the same cells. Therefore, the oligomeric forms may not be conserved between species, but rather may be associated with different functional states (Li et al. 2015b). *R. sphaeroides* TSPO (*Rs*TSPO) was found to be a monomer (Yeliseev and Kaplan 1995) or dimer (Yeliseev and Kaplan 2000; Li et al. 2013) depending on the growth conditions. Modeling evidence indicated a parallel dimer that was confirmed in several crystal structures and cryo-EM studies of *Rs*TSPO (Korkhov et al. 2010; Li et al. 2013, 2015c). The *B. cereus* TSPO was purified as both monomers and dimers but the form with the drug ligand PK11195 bound crystallized as a dimer (Guo et al. 2015), hinting that different ligands may bind to, or induce, different forms of TSPO. Mammalian TSPO has been reported to exist as monomer, dimer, and higher-order oligomeric forms (Yeliseev and Kaplan 1995; Lacapere et al. 2001; Batarseh et al. 2008; Teboul et al. 2012; Jaremko et al. 2014; Jaremko et al. 2015b; Issop et al. 2016; Zeng et al. 2018). The degree of oligomerization could be altered by treatment with tumor necrosis factor or hormones (Delavoie et al. 2003; Rone et al. 2009; Issop et al. 2016) and correlated with TSPO’s ability to bind different drug ligands or cholesterol (Gavish et al. 1992; Delavoie et al. 2003). Another report suggested that, while the TSPO monomer bound PK11195, benzodiazepine binding required a complex of TSPO and the voltage-dependent anion channel (VDAC) (Garnier et al. 1994b). Different binding partners most likely affect the oligomerization state and ligand binding specificity for mammalian TSPO. In plants, however, TSPO has been primarily reported to be a monomer. A monomer form of 18–22 kDa was found in *Arabidopsis thaliana* in a variety of tissues (Lindemann et al. 2004; Guillaumot et al. 2009a; Hachez et al. 2014; Jurkiewicz et al. 2018, 2020), although an early paper showed the presence of a possible dimer in potato (Corsi et al. 2004) and monomer and potential dimer forms were detected in *Digitalis lanata* cell cultures (Lindemann et al. 2004). More experimentation will be necessary to establish the potential for formation and functional significance of oligomeric forms in plants and other species.

### Location and orientation in membranes

TSPO’s cellular location and its orientation in membranes are two properties that have profound implications for potential binding partners and function. In bacteria, TSPO has been reported to localize in the outer cell membrane of *R. sphaeroides* (Yeliseev and Kaplan 1995), yet in the inner cell membrane of *Pseudomonas fluorescens* (Chapalain et al. 2009). A predicted orientation of *Rs*TSPO, based on mutation and crosslinking studies, was with the N-terminus facing outside of the cell and the first extramembrane loop facing the intermembrane space (Yeliseev and Kaplan 2000), whereas reports of the orientation of *Pseudomonas* TSPO varied (Chapalain et al. 2009; Leneveu-Jenvrin et al. 2014). In mammals, *Drosophila*, and the yeast *Schizosaccharomyces pombe*, TSPO is primarily located in the outer mitochondrial membrane (OMM) (Anholt et al. 1986; Verma and Snyder 1989; Papadopoulos et al. 1997, 2006; Kuhlmann and Guilarte 2000; Lin et al. 2014; Loth et al. 2020), an unsurprising location given the endosymbiotic origin of eukaryotic mitochondria. However, mitochondrial TSPOs were suggested to be inserted in a reverse orientation as compared to *Rs*TSPO, with the N-terminus facing the intermembrane space and the first loop facing the cytoplasm (Joseph-Liauzun et al. 1998). There are also reports of mammalian TSPO in the plasma membrane (Oke et al. 1992; Woods and Williams 1996; Woods et al. 1996) and nucleus (Kuhlmann and Guilarte 2000). In rat liver, TSPO was found in the OMM or plasma membrane in different subpopulations of cells (Woods et al. 1996). In glial cell lines that proliferated in response to TSPO ligands but did not make steroids, TSPO was reported to be nuclear, although it was mitochondrial in cells that made steroids but did not proliferate in response to ligands, leading Brown et al. (Brown et al. 2000) to suggest that TSPO’s location defined its function. A similar difference in location relative to proliferation was reported for liver tumor and breast cancer cells (Hardwick et al. 1999; Corsi et al. 2005). There is evidence that TSPO may localize to mitochondria-associated membranes (MAMs), zones where the endoplasmic reticulum (ER) contacts the OMM (McEnery et al. 1992; Gatliff et al. 2014; Zhou et al. 2015; Loth et al. 2020); to nucleus-associated mitochondria (NAMs) where mitochondrial and nuclear membranes meet (Desai et al. 2020); and to contact regions between the OMM and the inner mitochondrial membrane (IMM) (Lacapere and Papadopoulos 2003; Liu et al. 2006; Papadopoulos et al. 2006; Rone et al. 2012; Guilarte et al. 2016). Such contacts expand the range of possible binding partners for TSPO. The related mammalian isoform TSPO2 appears to be restricted to blood-forming tissues and to be located at multiple subcellular sites including mitochondria (Nakazawa et al. 2009), ER, nuclear membranes (Fan et al. 2009), and plasma membrane (Olson et al. 1988; Marginedas-Freixa et al. 2018; Manceau et al. 2020), which likely reflects the state of red blood cell differentiation.

In plants, TSPO has also been found in a variety of tissues (Corsi et al. 2004; Guillaumot et al. 2009a; Hachez et al. 2014) but its subcellular location is also controversial. TSPO was found in the ER and Golgi in *Arabidopsis* and a red alga (Guillaumot et al. 2009a; Vanhee et al. 2011b; Kobayashi et al. 2016), but also in mitochondria isolated from *Arabidopsis* (Balsemao-Pires et al. 2011) and *D. lanata* (Lindemann et al. 2004) and the nucleus of potato (Corsi et al. 2004). TSPO has also been found in chloroplasts from potato (Corsi et al. 2004), *Arabidopsis* (Balsemao-Pires et al. 2011), and *D. lanata* (Lindemann et al. 2004), as well as in cyanobacteria (Busch et al. 2017), the evolutionary forerunners of the chloroplast. *Arabidopsis* TSPO (*At*TSPO) was conclusively shown to be located in the ER/Golgi by fluorescent tagging of N-terminal 6-histidine-tagged *At*TSPO (Guillaumot et al. 2009a) and detection using antibodies raised to its N-terminal extension (Guillaumot et al. 2009a; Vanhee et al. 2011b); however, any N-terminally-shortened isoforms of *At*TSPO generated by alternative translation start codons (Lindemann et al. 2004; Balsemao-Pires et al. 2011; Cui et al. 2016) would not have been detected by using this method. Antibodies raised against amino acids 100–117 in the interior of the *At*TSPO protein showed TSPO to also be present in mitochondria (Lindemann et al. 2004). Using C-terminal GFP fusions instead of antibodies, Balsemao-Pires et al. (2011) detected full-length *At*TSPO in the ER under normal conditions but in chloroplasts under salt stress, while artificially-shortened forms of *At*TSPO were always mitochondrial. Jurkiewicz et al. (2020) recently suggested that the extended N-terminus was a unique plant evolutionary acquisition permitting ER localization to aid in the response to water stress. Thus, translation start sites and/or post-translational processing may determine plant TSPO localization (Cui et al. 2016) and therefore the range of possible binding partners.

Overall, it is clear that there are many questions concerning TSPO’s location and related function(s) in organisms of all kingdoms, questions that may only be answered by identifying specifically associated proteins.

## TSPO function

### Stress response and homeostasis

On the surface this ancient protein appears to have very different functions in different organisms, yet most of these functions relate to stress-induced changes in metabolism.

Mammalian TSPO has been associated with many physiological processes: cholesterol transport and steroidogenesis (Papadopoulos et al. 1997, 2006; Lacapere et al. 2001; Veenman et al. 2007; Rone et al. 2009), modulation of lipid metabolism (Thompson et al. 2013; Fan et al. 2015, 2020; Tu et al. 2016; Kim et al. 2019), autophagy (Gatliff et al. 2014, 2017; Kim et al. 2019; Moras et al. 2020), apoptosis (Carayon et al. 1996; Hirsch et al. 1998; Levin et al. 2005; Azarashvili et al. 2007, 2010; Veenman et al. 2007, 2008, 2014; Lin et al. 2014; Werry et al. 2015), alteration of Ca^2+^ signaling (Azarashvili et al. 2007; Ostuni et al. 2007), regulation of mitochondrial ATP production and respiratory control (Hirsch et al. 1989; Banati et al. 2014; Liu et al. 2017), generation/regulation of reactive oxygen species (ROS) (Lin et al. 2014; Gatliff and Campanella 2015; Guilarte et al. 2016), and heme/porphyrin regulation (Wendler et al. 2003; Papadopoulos et al. 2006). Human TSPO has also been implicated in diseases such as cancer [reviewed in (Gavish et al. 1992; Batarseh et al. 2010; Austin et al. 2013)], brain injury and neuroinflammation (Papadopoulos and Lecanu 2009; Liu et al. 2014), neurological diseases such as multiple sclerosis, Parkinson’s, Alzheimer’s, and Huntington’s (Gavish et al. 1999; Banati et al. 2000; Gerhard et al. 2006; Batarseh et al. 2010; Um and Yun 2017; Tournier et al. 2019), psychiatric disorders such as anxiety/panic disorder, depression, and bipolar disorder [reviewed in (Gavish et al. 1999; Rupprecht et al. 2010; Arbo et al. 2015)], and possibly even age-related macular degeneration (Wolf et al. 2020), obesity (Thompson et al. 2013; Fan et al. 2019), atherosclerosis (Gong et al. 2019; Kopecky et al. 2019), inflammatory bowel disease (Ostuni et al. 2010), diabetes (Fan et al. 2019), and HIV/AIDS (Zhou et al. 2014). One commonality among these associations was suggested to be a defense response to stresses caused by injury or disease (Veenman et al. 2007).

Plant TSPOs have also been reported to be involved in a wide variety of stress responses such as salinity (Kreps et al. 2002; Frank et al. 2007; Balsemao-Pires et al. 2011), osmotic stress (Kreps et al. 2002; Frank et al. 2007; Hachez et al. 2014; Jurkiewicz and Batoko 2018), cold (Kreps et al. 2002; Frank et al. 2007), pathogen invasion (Lehtonen et al. 2012), nutrient deficiency (Hermans et al. 2010), as well as regulation of porphyrin levels (Balsemao-Pires et al. 2011; Vanhee et al. 2011a; Kobayashi et al. 2016) and lipid metabolism (Jurkiewicz et al. 2018, 2020), and abscisic acid (ABA) signaling and responses (Frank et al. 2007; Guillaumot et al. 2009a; Vanhee et al. 2011a; Kobayashi et al. 2016; Jurkiewicz et al. 2020). Indeed, many functional associations of plant TSPOs may occur via response to ABA, a plant hormone that is upregulated by stress (Vanhee and Batoko 2011).

Microbial TSPOs also respond to stresses. In *R. sphaeroides*, TSPO has been described as part of a complex signaling pathway to switch between photosynthesis and respiration in response to oxygen levels, which involves regulation of a redox-sensing antirepressor of photosynthetic gene expression by modulating porphyrin export from the cell (Yeliseev and Kaplan 1995; Zeilstra-Ryalls et al. 1998; Oh and Kaplan 2001; Zeng and Kaplan 2001; Moskvin et al. 2007). Involvement of TSPO in light-regulated gene expression was suggested for the marine flavobacterium *Dokdonia* (Gonzalez et al. 2011) and the phototrophic bacterium *Dinoroseobactershibae* (Tomasch et al. 2011). In the cyanobacterium *F. diplosiphon*, TSPO was reported to be involved in adaptation of photosynthesis to light quality (Stowe-Evans et al. 2004; Busch et al. 2017), to regulate tetrapyrrole homeostasis, and in cellular responses to a variety of stresses, including regulation of oxidative stress (Busch and Montgomery 2015b; Busch et al. 2017) and in response to high salinity (Busch and Montgomery 2015a). In *Pseudomonas* Pf0-1, TSPO mRNA levels increased in response to high salt and hyperosmolarity and this protein was proposed to be part of a cell wall stress response (Leneveu-Jenvrin et al. 2014). TSPO also responds to nitrogen deprivation in *Sinorhizobium meliloti* (Davey and de Bruijn 2000) and *F. diplosiphon* (Busch et al. 2017).

TSPO’s general physiological function, conserved across all kingdoms, has been proposed to be maintenance of homeostasis (Gatliff and Campanella 2012; Batoko et al. 2015a; Gavish and Veenman 2018). For example, the moss *Pp*TSPO1 was suggested to be required for maintenance of redox homeostasis during the oxidative burst in response to fungal elicitor, possibly by its involvement in porphyrin metabolism (Lehtonen et al. 2012). In mammals, TSPO is upregulated in microglia during neuroinflammation yet had anti-inflammatory effects in those same cells, suggesting that TSPO expression was a response to limit inflammation (Bae et al. 2014) or maintain a balance between anti-inflammatory and pro-inflammatory mediators (Pozzo et al. 2019). In an insightful comparative review, Batoko et al.(2015b) concluded that TSPOs from bacteria, animals, and plants are required to modulate redox stress-related signaling and return cells to homeostasis. TSPO’s role in modulation of oxidative stress responses is a common theme [for reviews, see (Batoko et al. 2015a; Busch and Montgomery 2015b; Gatliff and Campanella 2015; Gavish and Veenman 2018)], and in this regard, porphyrins or lipids and steroids as TSPO ligands are frequently considered partners in control of cellular ROS levels. It is still unclear whether TSPO normally initiates stress responses (e.g., by increasing ROS production) or mitigates them (e.g., by controlling the level or duration of ROS production). TSPO may do both: initiate stress responses and subsequently reduce them to provide a tempered reaction to stress and maintain homeostasis. And despite extensive research demonstrating that TSPO binds several classes of small molecule ligands such as porphyrins, steroids, benzodiazepines, and other drugs [for reviews, see (Verma and Snyder 1989; Papadopoulos 1993; Chen et al. 2008; Batoko et al. 2015b; Veenman et al. 2016)], it remains unclear what the functional significance of these TSPO-ligand complexes is once they are formed.

### Receptor, sensor, transporter, or translocator?

Given the large disparity in the types of triggers and responses that have been reported to involve TSPO, one needs to dig deeply into cellular metabolism to find any links to a common mechanism of action for TSPO, if one exists. However, there are two direct demonstrations of conservation of function between bacteria, animals, and plants. The rat TSPO can functionally replace *Rs*TSPO in a *R. sphaeroides* knock-out mutant to restore the native function (Yeliseev et al. 1997), and adding the N-terminus of the plant *Arabidopsis* TSPO to mouse TSPO lets the mouse protein also bind the aquaporin PIP2;7 similarly to the native plant TSPO (Jurkiewicz et al. 2020). To be able to replace a TSPO in an evolutionarily-distant organism, TSPO must have some conserved aspects of its mechanism; yet to affect so many diverse cell processes, TSPO is most likely to be a regulatory protein or signal transducer that indirectly controls cell physiology.

It has been suggested that TSPO was originally a bacterial receptor/stress sensor that developed additional roles in eukaryotes (Zeng and Kaplan 2001; Fan et al. 2012; Li et al. 2016), which were themselves often stress-related. For example, the acquisition of an N-terminal extension in some plants has provided a unique way to respond to osmotic stress: as a selective autophagy receptor (Jurkiewicz et al. 2020) (see later). In most cases, it is unclear whether TSPO itself functions as a receptor/sensor, a transporter, or a translocator. Structural comparisons with G-protein-coupled receptors (GPCRs) and 10-transmembrane-helical transporters have suggested that TSPO could be similar to a GPCR-like receptor or, as a dimer, function as a translocator (Li et al. 2016). Mammalian and *R. sphaeroides* TSPOs contain a WxPxF motif (Fig. 1, purple box) predicted to serve as a flexible hinge and associated with a conformational change upon activation of GPCRs (Li et al. 2016). This motif lies near the proposed porphyrin binding site, suggesting that porphyrin binding could lead to conformational change and possibly alterations in oligomeric state or binding to partner protein(s) (Li et al. 2016). Most current evidence suggests that TSPO exerts it function(s) *indirectly* by association with its various protein binding partners, and binding of small molecules (such as porphyrins) may alter TSPO’s binding to its partners, thereby affecting its role in various metabolic processes.

## TSPO binding partners

It is reasonable to suggest that the evolution of new roles for TSPO is based on the acquisition of new interactions with new binding partners (Li et al. 2016). Here we discuss a number of proteins most commonly invoked as binding partners of TSPO (Fig. 2), even though in many cases a functional association with specific binding has not been completely established.

### Voltage-dependent anion channel and the adenine nucleotide transporter

Perhaps the most widely researched interaction of TSPO is that with the voltage-dependent anion channel (VDAC), a channel in the mammalian OMM that controls a wide range of processes involving transport of molecules into and out of mitochondria [recently reviewed in (Shoshan-Barmatz et al. 2019)]. VDAC1 (one of 3 isoforms) is the major form expressed in most mammalian cells and its functional interaction with TSPO is the best-studied, as both are associated with mitochondrial processes such as cholesterol import, mitophagy, regulation of cytosolic Ca^2+^ levels, ROS generation, apoptosis, and regulation of the mitochondrial permeability transition pore (MPTP) (Gatliff and Campanella 2016; Shoshan-Barmatz et al. 2019). Some human diseases such as Parkinson’s and Alzheimer’s are associated both with TSPO (Gavish et al. 1999; Batarseh et al. 2010; Tournier et al. 2019) and with VDAC (Kanwar et al. 2020), suggesting an interplay between these partners in disease states.

**Fig. 3:**
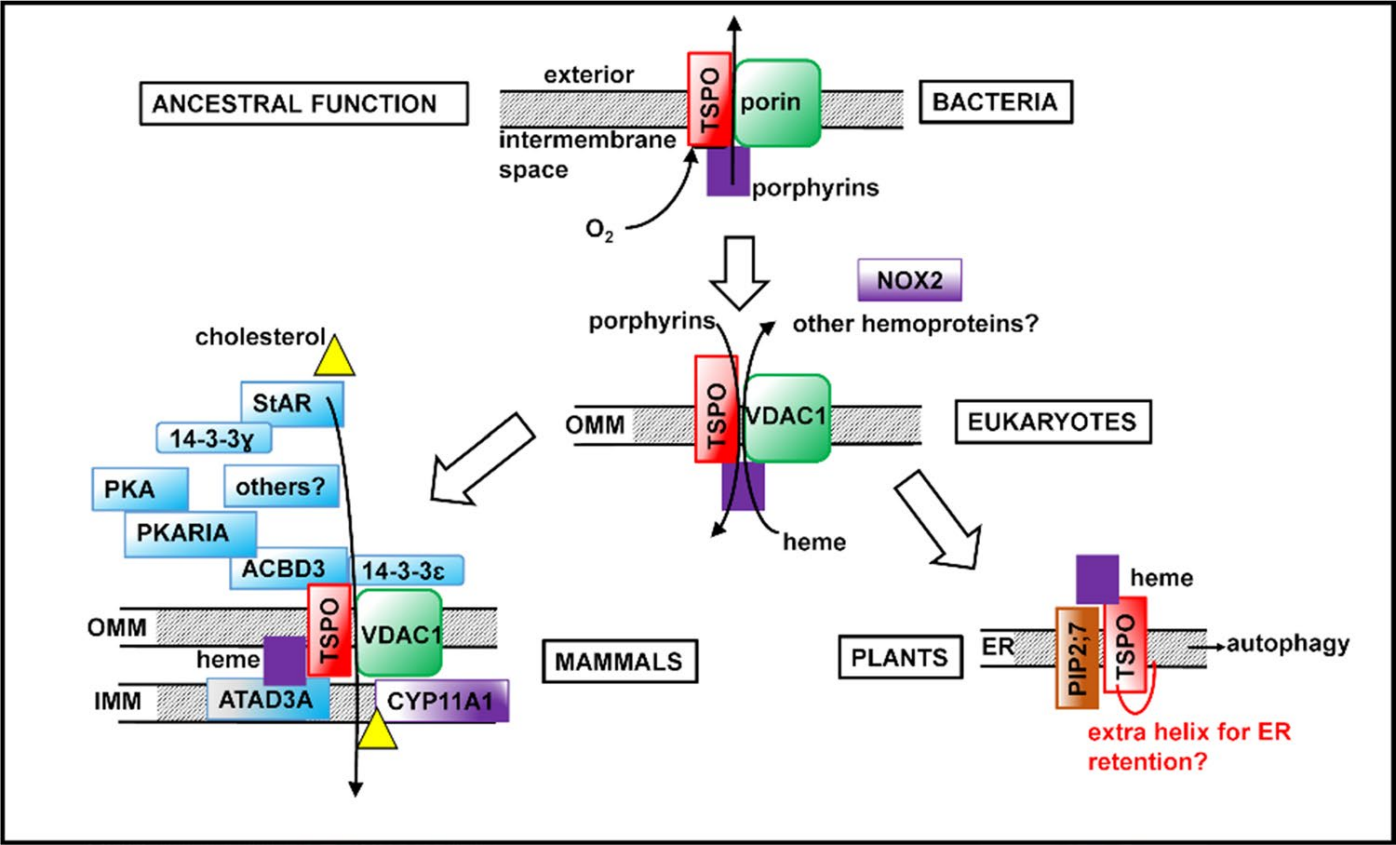
Evolution of TSPO functions. TSPO’s earliest function in porphyrin export from bacterial cells may have evolved into import of PPIX into and export of heme out of mitochondria and chloroplasts in eukaryotes. Functions acquired later in evolution include functioning as a SAR in plants and participating in a large protein complex that imports cholesterol into mitochondria of steroidogenic tissues in mammals. Purple squares denote porphyrins; yellow triangles denote cholesterol. Proteins involved in cholesterol transport are shaded blue; heme-containing proteins are shaded purple

A role for TSPO in the modulation of steroid metabolism in animals is supported by considerable data. TSPO was long thought to be an important player in the transport of cholesterol into mitochondria, a rate-limiting step in steroid hormone biosynthesis [reviewed in (Papadopoulos et al. 2006; Veenman et al. 2007; Rone et al. 2009)]. Mammalian TSPO binds cholesterol with nanomolar affinity (Lacapere et al. 2001), and this tight binding involves a cholesterol recognition/interaction amino acid consensus (CRAC) motif in the C-terminal part (Li and Papadopoulos 1998; Lacapere and Papadopoulos 2003) and was observed to be augmented by a nearby leucine-alanine-phenylalanine (LAF) binding enhancement motif (Li et al. 2015a) (Fig. 1, red and blue boxes). An early review suggested that TSPO functioned as a channel to bring cholesterol into mitochondria and did not require any binding partners (Veenman et al. 2007). However, none of the TSPO crystal structures provide any obvious clues regarding channels for cholesterol transport, either within a TSPO monomer or at the dimer interface (Guo et al. 2015; Li et al. 2015c). Most recent evidence suggests that TSPO is likely not in itself a transporter for cholesterol, but rather is involved in multi-protein complexes that translocate or regulate cholesterol distribution (Li et al. 2016) (Fig. 3, bottom right). One such 800 kDa complex included TSPO and VDAC in the OMM, and ATPase family AAA domain-containing protein 3A (ATAD3A) and the cytochrome P450 enzyme CYP11A1 in the IMM (Liu et al. 2006; Rone et al. 2012). Cholesterol could be bound between TSPO and VDAC, as the CRAC site in TSPO is oriented to be able to interact with the lipid membrane or with the membrane-buried VDAC to create a cholesterol binding site (Li et al. 2016). Curiously, plant mitochondrial VDAC can be regulated by plant sterols in a manner that involves a protein-sterol interaction but not sterol-induced alteration of membrane properties (Mlayeh et al. 2010). Further investigation of TSPO’s role in this regulation is warranted.

In animals, TSPO has been linked with apoptosis, by virtue of its interactions with VDAC and by its effects on ROS. VDAC and the adenine nucleotide transporter (ANT) were originally hypothesized to form the core of the MPTP required for apoptosis, with its function being modulated by further association with TSPO (Veenman et al. 2008) since these three proteins were observed to copurify (McEnery et al 1992). However, the MPTP can form when ANT, VDAC, or TSPO are individually absent (Kokoszka et al. 2004; Baines et al. 2007; Sileikyte et al. 2014). A study of rat liver mitochondria versus mitoplasts demonstrated that the OMM was not required for MPTP formation but played a regulatory role, and the authors suggested TSPO could have a dual function as a regulator of the permeability transition and as a transporter of MPTP-active molecules to their binding sites in the IMM or matrix (Sileikyte et al. 2011). Thus, TSPO may be a non-essential part of the MPTP, but modulation of apoptosis by TSPO and VDAC cannot be ruled out. TSPO and VDAC have been implicated in bacterially-induced apoptosis caused by treatment of rat glial cells with *Pseudomonas fluorescens* MF37, as this process was significantly reduced when the bacteria were pre-treated with the TSPO ligand PK11195 and this reduction required the bacterial outer membrane porin OprF (analogous to VDAC) (Chapalain et al. 2009). Although plants can undergo programmed cell death (the hypersensitive response) in response to pathogens, plant genomes lack orthologs of most mammalian apoptosis regulators (Williams and Dickman 2008) and so far no reports have linked plant TSPO and VDAC to programmed cell death or apoptosis.

Direct binding of the TSPO protein to VDAC and ANT was first reported in 1992 when these proteins co-purified with TSPO from kidney mitochondria (McEnery et al. 1992). In an early study of mouse Leydig cells using transmission electron microscopy and atomic force microscopy, the structures seen were consistent with clusters of 4–6 TSPO molecules and one VDAC (Papadopoulos et al. 1994). TSPO from digitonin-solubilized mitochondrial extracts eluted on gel filtration columns as 170 to 240 kDa complexes containing TSPO, VDAC, ANT, and some unidentified proteins at ~ 60 kDa (Rone et al. 2009). Cross-linking with photoactivatable amino acids in COS cells (kidney fibroblasts) and hormone-treated Leydig cells (testicular steroidogenic cells) showed a complex of steroidogenic proteins designated as the “transduceosome” in the OMM (Rone et al. 2009). This complex contained TSPO, the PBR-associated protein 7 (PAP7; also called the Golgi complex-associated protein 60, but recently renamed acyl-CoA binding domain containing 3 or ACBD3), the regulatory subunit RIα of protein kinase A (PKARIA), and VDAC (Rone et al. 2009). Further immunoblot and mass spectrometry analyses by these authors revealed an 800 kDa complex that contained TSPO and VDAC along with several IMM proteins but not ANT (Rone et al. 2012). By using anti-his-tag antibodies, VDAC1 could be co-immunoprecipitated with a his-tagged TSPO from mouse embryonic fibroblasts (MEFs) and the CRAC motif of TSPO was not required for this TSPO-VDAC1 interaction (Gatliff et al. 2014). In red blood cells, however, TSPO ligands stimulated ATP release by a complex involving VDAC, TSPO2 and ANT (Marginedas-Freixa et al. 2018). The most recent evidence suggests that TSPO probably does not directly bind to ANT, although it may interact via VDAC or indirectly by regulation of some metabolic processes that involve ANT. Several studies have shown that the expression levels of TSPO and VDAC1 are oppositely regulated in mammals; for example, overexpression of TSPO reduced VDAC1 levels and siRNA knock-down of TSPO increased VDAC1 expression in endothelial cells (Joo et al. 2012). In contrast, down‐regulation of VDAC1 in glioblastoma cells also down‐regulated TSPO expression (Arif et al. 2016). Ligand-binding studies have shown a functional connection between TSPO and regulation of complexes containing TSPO and VDAC. In MEFs and canine tumor cell lines, TSPO formed a complex with VDAC, protein kinase A (PKA), and ACBD3 in which VDAC1 function was dependent upon TSPO-regulated phosphorylation of VDAC1 by PKA (Gatliff et al. 2017). In red blood cells, TSPO2 and VDAC were localized to membrane microdomains together (Marginedas-Freixa et al. 2016) and TSPO ligands were found to bind to the TSPO2-VDAC complex to stimulate the uptake of Zn protoporphyrin IX (ZnPPIX) and increase ROS accumulation (Marginedas-Freixa et al. 2016). The TSPO drug ligands Ro5-4864, NCS1018, and TRO19622 also induced polymerization of VDAC, coupled to activation of ATP release by a supramolecular complex involving VDAC, TSPO2 and adenine nucleotide transporter (ANT) (Marginedas-Freixa et al. 2018). A computational pathway analysis of interacting proteins required to initiate apoptosis predicted that TSPO would interact with VDAC1 and VDAC3 directly and VDAC2 indirectly (Shailaja et al. 2019). This is of interest for TSPO’s function in apoptosis, as VDAC2 was the form reported to be required for apoptosis and VDAC2-knockout mice are embryonic lethal (Cheng et al. 2003); however, VDAC1/3-knockout MEFs, VDAC2-knockout MEFs and VDAC1/3-knockout + VDAC2-knockdown MEFs had enhanced cell death but still could form the MPTP (Baines et al. 2007). Further studies are needed to address which forms of VDAC are involved in mammalian TSPO-related functions.

In plants, the only report of a physical association of TSPO and VDAC was in Vanhee et al. (2011b). When the *Arabidopsis* TSPO was expressed in yeast, subcellular fractionation experiments showed that *At*TSPO co-sedimented with VDAC1, demonstrating that VDAC1 and *At*TSPO were co-localized in the same membrane (Vanhee et al. 2011b). Affinity purification of a histidine-tagged *At*TSPO expressed in yeast resulted in co-purification of some VDAC1, while anti-VDAC1 antibodies co-immunoprecipitated *At*TSPO and anti-TSPO antibodies co-immunoprecipitated VDAC1, demonstrating a physical interaction between *At*TSPO and yeast VDAC1 (Vanhee et al. 2011b). In the bacterium *P. fluorescens* MF37, a functional relationship between TSPO and the major outer membrane porin OprF (corresponding to the mammalian VDAC) was shown by the abolishment of the biological effects of PK11195 in a OprF deletion mutant (Chapalain et al. 2009). But since the *P. fluorescens* TSPO was reported to be in the inner cell membrane, a physical associated with porin was discounted despite in silico prediction of a functional association (Leneveu-Jenvrin et al. 2014). While Yeliseev and Kaplan (1995) speculated that *Rs*TSPO could physically interact with the porin, no direct binding between the two proteins was investigated in *R. sphaeroides*.

In general, the evidence supporting a TSPO/VDAC interaction is substantial, but the functional significance of that interaction remains an important question in multiple species.

### Steroidogenic acute regulatory protein

The mammalian steroidogenic acute regulatory (StAR) protein is a hormone-induced, mitochondria-targeted protein that shuttles ER-derived cholesterol from the cytoplasm to the mitochondria in a PKA-dependent manner (Herrera-Cruz and Simmen 2017), possibly at MAMs (Vance 2014). StAR has been proposed to interact with TSPO at the OMM to facilitate cholesterol import (Hauet et al. 2005; Liu et al. 2006) as part of a large signaling complex involving TSPO, ACBD3, and PKARIA (Liu et al. 2006) (Fig. 3, bottom left). Evidence of a functional connection of TSPO to StAR was found in TSPO-deficient cells, in which StAR levels were increased and the mitochondrial membrane potential was reduced (Fan et al. 2018). In TSPO-depleted cells, StAR was not processed from its 37 kDa cytosolic size to the mature intramitochondrial 30 kDa size (Hauet et al. 2005; Rone et al. 2009), although the converse experiment, a knock-down of StAR, did not affect TSPO (Hauet et al. 2005). TSPO was suggested to play a direct role in the import of StAR into the IMM (Rone et al. 2009), or an indirect role via regulation of the mitochondrial membrane potential (Fan et al. 2018). It has also been suggested that StAR could transfer cholesterol to TSPO at the OMM, since StAR’s affinity for cholesterol is much lower than TSPO’s ~ 5 nM affinity (Liu et al. 2003; Rone et al. 2009). However, TSPO knockdown in mouse and human steroidogenic cell lines did not affect steroidogenesis or StAR expression (Tu et al. 2014) and in TSPO knockout mice, no significant differences were seen in the expression levels of StAR, VDAC1, or ATAD3A, members of the mitochondrial transduceosome complex (Fan et al. 2015). StAR and TSPO were shown in one study to associate at the mitochondrial membrane within at least 100 angstroms of each other as demonstrated by FRET (fluorescence resonance energy transfer) (West et al. 2001). In another study, however, there was no evidence of direct StAR/TSPO interactions either by BRET (bioluminescence resonance energy transfer) or by bacterial and mammalian two-hybrid assays (Bogan et al. 2007). Photoaffinity-labeled TSPO has been reported to form several high molecular weight complexes, but these complexes did not include StAR (Bose et al. 2008). Most current evidence suggests that the TSPO-StAR interaction is indirect, most likely through other TSPO binding partners such as VDAC or ACBD3.

StAR belongs to a large family of START (steroidogenic acute regulatory protein-related lipid-transfer) motif-containing proteins. StAR itself has not been found in plants or bacteria, but many more genes encoding START family proteins have been found in plants than in animals (Soccio and Breslow 2003) and putative START domains have been found in genes from a few bacterial genera and unicellular protists (Schrick et al. 2004). Currently, there are no reports of any connections between these START proteins and TSPO.

### Kinases and kinase-anchoring proteins

Evidence of TSPO phosphorylation has been reported (Whalin et al. 1994; Batarseh et al. 2010; Chen et al. 2014; Wang et al. 2021) and TSPO has been functionally linked with Ca^2+^-dependent protein kinase C (PKC) and cAMP-dependent protein kinase A (PKA) via its regulatory subunit Iα (PKARIA). These kinases are attached to the mitochondrial membrane by anchoring proteins (such as A-kinase anchoring proteins or AKAPs) and may influence steroid and lipid metabolism and ATP signaling through phosphorylation of VDAC and possibly TSPO.

TSPO2 is expressed in mammalian red blood cells and the TSPO ligands Ro5-4864, NCS1018, and TRO19622 induce VDAC dimerization and the release of ATP via an increase in the cAMP content (Marginedas-Freixa et al. 2018). In these cells, PKA is activated by the β-adrenergic pathway and TSPO2-ligand-activated ATP release is inhibited by addition of a PKA inhibitor, a PKC inhibitor, or 2-methyl-S-ADP (used to decrease the cAMP content), demonstrating that PKA and PKC can modulate TSPO2-induced ATP release (Marginedas-Freixa et al. 2018). Triggering an increase in extracellular ATP is a fundamental signaling process involved in damage control and inflammation (Di Virgilio et al. 2020), processes with which TSPO has been associated. No direct binding of TSPO2 to either kinase was demonstrated, suggesting the interaction could be indirect and possibly via VDAC. However, phosphorylation motifs have been found in the C-terminal domain of mouse, rat, and bovine (but not human) TSPO1 (Whalin et al. 1994; Batarseh et al. 2010). PKA (but not other kinases) was able to phosphorylate mouse TSPO (Whalin et al. 1994; Batarseh et al. 2010) and PKA activation was necessary for TSPO phosphorylation in rat hypothalamic astrocytes in response to estradiol (Chen et al. 2014). This suggests that direct binding of specific kinases to specific TSPOs could be a method of regulation by enhancing phosphorylation of TSPO or its binding partners.

A yeast two-hybrid screen of a mouse testis cDNA library, using either TSPO or PKARIA as bait, revealed ACBD3 (acyl-CoA binding domain-containing protein 3; also known as PAP7), a ~ 60 kDa protein located in mammalian Golgi and mitochondria and possibly the ER (Li et al. 2001; Liu et al. 2003; Fan et al. 2010). ACBD3 has been proposed to function as an AKAP by anchoring PKARIA and thereby targeting PKA to mitochondria, where it could phosphorylate StAR and promote cholesterol transfer from the low affinity StAR to the high affinity TSPO (Liu et al. 2003; Fan et al. 2010). Immunohistochemical and in situ hybridization studies indicated that, like TSPO, ACBD3 is highly expressed in steroidogenic tissues, and hormone-induced steroidogenesis in MA-10 Leydig cells was increased by overexpression of ACBD3 and inhibited by antisense knockdown of ACBD3 (Li et al. 2001). Stress hormone treatment induced colocalization of TSPO, ACBD3, PKARIA, and StAR in mitochondria (Liu et al. 2006). Cross-linking by photoactivatable amino acids has shown a large protein complex in the OMM containing TSPO, ACBD3, PKARIA, and VDAC (Rone et al. 2009). Co-immunoprecipitation and western blotting demonstrated that ACBD3 bound directly to TSPO and PKARIA (Li et al. 2001). Steroidogenesis was inhibited by disruption of the PKARIA-ACBD3 and ACBD3-TSPO interactions by using either ACBD3 mutants or the peptide Ht31 known to disrupt the PKA anchoring (Liu et al. 2006). A recent report suggested that the drug G-1 may ease anxiety by activating a G-coupled estrogen receptor in the central nervous system to promote release of cAMP and thereby activate PKA, which increases the phosphorylation of TSPO, which then regulates neurosteroidogenesis (Wang et al. 2021).

ACBD3 and AKAPs have been linked with TSPO in other processes besides steroidogenesis. Increased TSPO levels caused ROS generation in MEFs, which was attributed to increased formation of a complex of PKA and ACBD3 with TSPO and VDAC (Gatliff et al. 2017). ACBD3 is upregulated by photodynamic therapy, which is possibly mediated by its interaction with TSPO (Fan et al. 2010). A newly-reported interaction, nucleus-associated mitochondria (NAM), has been identified as part of a mitochondrial retrograde signaling pathway in response to the mitochondrial stressor staurosporine, in which a complex of TSPO, PKA, ACBD3, and AKAP95 was required to tether mitochondria to the nucleus (Desai et al. 2020). ACBD3 contains an acyl-CoA binding motif similar to the one identified in the diazepam binding inhibitor (DBI; see below), suggesting they could share a common site of interaction with TSPO (Lacapere and Papadopoulos 2003; Liu et al. 2003; Rone et al. 2009).

TSPO expression was shown to be under the control of a PKCɛ-related transcriptional signaling pathway (Batarseh et al. 2008, 2010; Gatliff et al. 2014) and PKCɛ has been suggested to be a “master regulator of TSPO expression and function” (Batarseh et al. 2010). No direct binding of PKC to TSPO was shown, but PKCɛ was co-immunoprecipitated with VDAC and ANT from mouse heart mitochondria (Budas and Mochly-Rosen 2007), suggesting a possible indirect interaction with TSPO via VDAC. The indirect functional interaction may also be via ROS: when an activator of PKC was used to induce vascular endothelial cell activation, ROS was produced and this ROS production was inhibited by TSPO overexpression and enhanced by siRNA-silencing of TSPO (Joo et al. 2015).

Plants do not have protein kinase C (Jurkiewicz et al. 2020) and thus far plant TSPO has not been associated with other protein kinases. However, some functional connections to phosphatidylinositol kinases have been suggested. In *Arabidopsis*, TSPO degradation was sensitive to inhibitors of type III phosphoinositide 3-kinases, which regulate autophagy in eukaryotic cells (Vanhee et al. 2011a). The level of *At*TSPO was increased 6- to eightfold after treatment of *Arabidopsis* seedlings with a phosphatidylinositol-3-kinase inhibitor or with an autophagosome formation inhibitor that promotes phosphatidylinositol-3-kinase complex degradation (Hachez et al. 2014). Jurkiewicz et al. suggested that expression of *At*TSPO induces phosphatidylinositol-4,5-bisphosphate depletion in the plasma membrane and its enrichment in the Golgi membrane by enhancing phosphorylation of its precursor phosphatidylinositol-4-phosphate within the Golgi (Jurkiewicz et al. 2020). However, these connections between phosphatidylinositol kinases and TSPO may be indirect and not due to direct protein–protein binding.

### 14-3-3 proteins

The 14-3-3 proteins are small (about 30 kDa), conserved, acidic proteins found in all eukaryotes. They function as dimers and act as scaffolds because each monomer in the dimer can bind independently to different target proteins (Chevalier et al. 2009; Ballone et al. 2018). All 14-3-3 isoforms (including β, γ, ζ, ɛ, η, ϭ, and ɵ in mammals) contain an N-terminal dimerization domain and a C-terminal target binding domain, and their targets are isoform-specific (Chevalier et al. 2009; Aghazadeh et al. 2014; Ballone et al. 2018).

The presence of 14-3-3 binding motifs have been found in the sequences of animal TSPO (Fig. 1, yellow box), StAR, ABCD3, PKARIA, and VDAC (Aghazadeh et al. 2012). By using photoactivatable amino acids to crosslink 14-3-3γ and its targets, Aghazadeh et al. (2012) showed in cultured Leydig cells that 14-3-3γ binds to TSPO, VDAC, PKARIA, ACBD3, and 14-3-3ζ, while co-immunoprecipitation experiments showed that the interaction of 14-3-3γ with StAR required cAMP treatment (Aghazadeh et al. 2012). These researchers suggested that 14-3-3γ was a member of or played a role in the assembly of the transduceosome complex (Aghazadeh et al. 2012). By using a combination of co-immunoprecipitation and cross-linking by photoactivatable amino acids, TSPO, VDAC1, and StAR were shown to interact with the 14-3-3ε isoform in a cAMP-dependent manner (Aghazadeh et al. 2014). Mutated peptides mimicking regions of VDAC1 proposed to be part of the 14-3-3ε binding site were used to disrupt the VDAC1-14-3-3ε interaction, which inhibited binding of StAR to 14-3-3ε but increased the TSPO-VDAC1 interaction, suggesting that the 14-3-3ε scaffold intercalates between TSPO and VDAC1 (Aghazadeh et al. 2014). A complex regulatory pathway was proposed: 14-3-3ε and StAR associate in the cytosol; VDAC1 then competes off StAR to bind to 14-3-3ε, resulting in relocalization of 14-3-3ε to mitochondria and its intercalation between TSPO and VDAC1. This pathway is proposed to result in reduced cholesterol import into mitochondria and downregulation of steroidogenesis (Aghazadeh et al. 2014). Later publications proposed a more complex regulation involving 14-3-3γ as well as 14-3-3ε (Aghazadeh et al. 2015; Aghazadeh and Papadopoulos 2016). In both cases 14-3-3ɛ is proposed to intercalate between TSPO and VDAC and reduce the rate of cholesterol import and slow steroidogenesis. Different isoforms of 14-3-3 may therefore bind to TSPO after different stimuli, such as cAMP or hormones, and thereby alter its activity or function by regulating its interaction with VDAC.

In plants, 14-3-3 proteins have been shown to regulate a wide variety of processes including several metabolic pathways, transcription control, phosphorylation-related signal transduction, and responses to pathogen attack and abiotic stress, and have been found in chloroplasts and mitochondria (Chevalier et al. 2009). Although some of their reported functions overlap with processes in which TSPO has been implicated (e.g., ABA responses), there are no reports linking 14-3-3 proteins to plant TSPO. The 14-3-3 family of proteins do not appear to be present in prokaryotes (Chevalier et al. 2009). The evolution of this protein family in eukaryotes may have provided TSPO access to more binding partners or altered the binding of TSPO to its old partners (such as VDAC/porin) and thereby promoted new TSPO-associated functions.

### Diazepam binding inhibitor/acyl-CoA binding protein/acyl-CoA binding domain protein 1

In 1977, an endogenous peptide that lowered diazepam affinity for rat brain GABA receptors in neuronal membranes, was discovered and called the diazepam binding inhibitor (DBI) (Braestrup et al. 1977), while the bovine ortholog was isolated independently and called endozepine (Farzampour et al. 2015; Tonon et al. 2020). Later, a protein capable of binding acyl-CoA esters was found and called the acyl-CoA-binding protein (ACBP) (Tonon et al. 2020). All three would turn out to be the same ~ 10 kDa cytosolic protein, part of a large family of acyl-CoA binding domain (ACBD) proteins with a conserved acyl-CoA binding (ACB) domain consisting of 4 α-helices arranged in a bowl shape [reviewed in (Islinger et al. 2020)]. DBI was therefore also denoted as ACBD1. ACBD proteins were found to be expressed in many animal tissues but primarily in steroidogenic tissues such as adrenal cortex, testis, and liver (Bovolin et al. 1990; Lacapere and Papadopoulos 2003), as well as tissues associated with fatty acid turnover such as adipose tissue (Nitz et al. 2005), and in the brain (Malagon et al. 1993; Tonon et al. 2020). They were shown to bind saturated and unsaturated C14-C22 fatty acyl-CoA esters and have many functions related to long-chain fatty acyl-CoAs including: stabilization, prevention from partitioning into membranes, extraction from membranes, transfer to their metabolizing enzymes, or control over their regulatory effects on lipid-metabolizing proteins or lipid-binding receptors [reviewed in (Islinger et al. 2020)].

DBI, the smallest ACBD protein (~ 10 kDa), has long been cited as an endogenous TSPO ligand in animals (Guidotti et al. 1983; Papadopoulos et al. 1991, 1992; Papadopoulos 1993; Rone et al. 2009). DBI is reported to have nanomolar affinity for TSPO (Papadopoulos 1993). The locations of DBI mRNA and TSPO in peripheral tissues correlate well (Tonon et al. 2020). Naturally processed peptides of DBI, the octadecaneuropeptide (ODN; amino acids 33–50) and the triakontatetraneuropetide (TTN; amino acids 17–50) (Slobodyansky et al. 1989) have been found to bind to TSPO in the brain, adrenals, and testis (Papadopoulos 1993; Lacapere and Papadopoulos 2003). DBI and TTN were reported to functionally interact with TSPO to stimulate steroidogenesis (Papadopoulos et al. 1991). It was later shown that both DBI and TTN (but not ODN) could displace benzodiazepines from TSPO and stimulate cholesterol transfer into mitochondria of steroidogenic cells (Papadopoulos et al. 1992; Papadopoulos 1993; Lacapere and Papadopoulos 2003). This suggests that DBI/ACBD1, as well as ACBD3, could be involved in the regulation of cholesterol transport into mammalian mitochondria. DBI was localized to the ER, Golgi, and OMM in rat testis (Schultz et al. 1992) where it would have the opportunity to bind to TSPO, and chemical crosslinking studies demonstrated that DBI binds directly to TSPO (Garnier et al. 1994a). The interaction between DBI and TSPO has been deemed critical for hormone stimulation of adrenal steroidogenesis [reviewed in (Fan et al. 2010)], and most of the reported connections between TSPO and DBI involve this metabolic process. In addition, both TSPO and DBI are upregulated in a mouse model of retinal inflammation and injury (Wang et al. 2014), suggesting that their interconnection may not be exclusive to steroidogenesis.

Relative to TSPO, however, there are complications for interpreting results involving DBI and its peptide products. These peptides can interact with the neuronal plasma membrane GABA-A receptor as well as TSPO, and some effects of ODN were reported to be mediated through an unidentified GPCR coupled to the phospholipase C/PKC and/or adenylyl cyclase/PKA pathways (Tonon et al. 2020). DBI and ODN have been reported to stimulate neurosteroid synthesis and neurogenesis via GABA-A receptor modulation (Islinger et al. 2020), which could complicate interpretation of investigations of neurological functions if DBI binding is used to confirm TSPO’s role. The concentration of DBI used is likely to be important, as some authors have reported that DBI binds to the GABA-A receptor but with low affinity (Rone et al. 2009; Tonon et al. 2020); other authors have stated that direct binding of DBI to the GABA-A receptor has never been shown (Islinger et al. 2020). TSPO’s role in experiments on autophagy also need confirmation by other means than DBI binding. Although TSPO knockdown or DBI knockdown similarly inhibited autophagy (Bravo-San Pedro et al. 2019), DBI can also be secreted through a Golgi-dependent pathway (Charmpilas et al. 2020) and, once outside of the cell, it may inhibit autophagy via the plasma membrane GABA-A receptor (Bravo-San Pedro et al. 2019).

In plants, ACBD proteins have been found in a wide range of species (e.g., *Brassica napus*, cotton, castor bean, rice, grape, *Agave americana*, *D. lanata*) and have been associated with a wide range of functions, including fatty acid metabolism, freezing tolerance, pathogen resistance, embryogenesis, seed germination and seedling development, oxidative stress, and hypoxia [reviewed in (Du et al. 2016)]. Although a *D. lanata* ACBD protein could displace PK11195 from mouse Leydig cell mitochondria (Lindemann et al. 2004), plant ACBD proteins have not been reported to bind to any plant TSPO and none of the functions ascribed to plant ACBD proteins have been shown experimentally to involve TSPO. Nevertheless, a few of the plant ACBD proteins have been associated with processes with which TSPO has also been associated, hinting at potential functional, if not necessarily physical, connections. *Arabidopsis At*ACBP2-overexpressing plants showed improved drought tolerance and upregulation of genes encoding two NADPH oxidases essential for ABA-mediated ROS production (Du et al. 2013, 2016). TSPO has been invoked in one case in the regulation and generation of ROS in mammals by providing the heme cofactor for NADPH oxidase assembly (Guilarte et al. 2016; Loth et al. 2020) (see next section); a similar indirect role could apply in plants. *At*ACBP3, which was localized to the extracellular space, intracellular membranes, and the ER/Golgi, promoted degradation of ATG8 and disrupted autophagosome formation when overexpressed (Xiao et al. 2010). *At*TSPO is also mainly ER/Golgi localized (Guillaumot et al. 2009a), physically interacts with ATG8 (Hachez et al. 2014), and is mainly degraded via autophagy (Vanhee et al. 2011a) (see later), so the interaction of *At*TSPO and *At*ACBD3 in plant autophagy needs further investigation. All *At*ACBPs are highly expressed in developing seeds (Du et al. 2016), as is *At*TSPO (Guillaumot et al. 2009a). Like TSPO, ACBPs are associated with plant stress responses. A cytosolic ACBD protein from the alga *Chlorella* sp.JB6 was upregulated under various stresses including NaHCO_3_, NaCl, H_2_O_2_, CuCl_2_, and cold (Qiao et al. 2018), and similar upregulation of ACBD proteins was documented in response to salt stress in cotton (Qiao et al. 2018), to H_2_O_2_ stress in *Arabidopsis* (Gao et al. 2009), and to heavy metals in *Arabidopsis* (Xiao et al. 2008; Gao et al. 2009; Qiao et al. 2018). (Note that mammalian DBI can bind lead with nM affinity (Smith et al. 1998).) The closest homolog to the mammalian DBI/ACBD1 in *Arabidopsis*, *At*ACBD6 (Xiao and Chye 2011), was cold-induced and plants overexpressing *At*ACBP6 were more resistant to freezing (Chen et al. 2008). This freezing resistance was correlated with upregulation of phospholipase Dδ (PLDδ) and loss of phospholipids (Chen et al. 2008). TSPO is also associated with many stress responses: salt stress in *Arabidopsis* (Guillaumot et al. 2009a; Balsemao-Pires et al. 2011), the red alga *C. merolae* (Kobayashi et al. 2016), the cyanobacterium *F. diplosiphon* (Busch and Montgomery 2015a; Busch et al. 2017), and the eubacterium *Pseudomonas* Pf0-1 (Leneveu-Jenvrin et al. 2014); and oxidative and cold stress responses in moss (Frank et al. 2007; Lehtonen et al. 2012) and *F. diplosiphon* (Busch et al. 2017). Thus, investigation of the co-regulation of both TSPO and ACBD proteins in salt, cold, or oxidative stresses could be fruitful. However, the same caveat for researching the interaction of TSPO and ACBD proteins in mammals will likely also apply to plants, as in at least one case, a plant ACBD protein has been shown to bind to a protein partner other than TSPO; in this case, plasmodesmata-localized protein 8 (Ye et al. 2017).

The ACB domain has been postulated to be a very ancient protein structure (Islinger et al. 2020) and small ACBD proteins carrying only an ACB domain are also found in eubacteria and archaea (Burton et al. 2005; Islinger et al. 2020). Islinger et al. suggested that a common ancestor of ACBD proteins may have already been present before the evolution of the first eukaryotes (Islinger et al. 2020). An earlier report suggested that many of the prokaryotic ACBD proteins were present in pathogenic bacteria and that ACBD genes could have been acquired from eukaryotic hosts by horizontal gene transfer (Burton et al. 2005). Prediction of the potential structures of prokaryotic ACBD sequences showed that they could form typical 4 helix bundles and maintain the hydrophobic and hydrophilic surface patches that are seen in eukaryotic ACBD proteins (Islinger et al. 2020), suggesting that they could have functions similar to their roles in eukaryotes. However, to date there are no reports of any connections between prokaryotic ACBD proteins and TSPO.

### NADPH oxidase

NADPH oxidase (NOX) is a heme protein and major producer of oxygen radicals. The synthesis of NOX has been proposed to involve heme donation by TSPO (Fig. 3, center). Exposure of primary microglia to the TSPO ligands PK11195 or Ro5-4864 increased microglia proliferation and production of ROS; this ROS production was abrogated by NOX isoform 2 (NOX2) inhibitors (Choi et al. 2011). NOX2 is a major source of ROS in the central nervous system. Endogenous TSPO ligands such as TTN released from activated astrocytes may stimulate TSPO to transfer heme from mitochondria, where it is produced, to the ER to support NOX2 synthesis (Guilarte et al. 2016). Production of ROS by NOX2 can activate nuclear factor erythroid 2 related factor 2 (Nrf2), a transcription factor that regulates over 600 cytoprotective genes involved in redox homeostasis (Guilarte et al. 2016).

A functional connection between TSPO and NOX2 was shown in mammalian phagocytes. The levels of TSPO paralleled the levels of one of the NADPH oxidase subunits (gp91^phox^) in neutrophils from two types of chronic granulomatous disease patients, one with a deficiency in gp91^phox^ and decreased TSPO and the other with normal levels of both proteins (Zavala et al. 1990). A monoclonal antibody against TSPO enhanced NOX2 activity (the oxidative burst) in neutrophils (Zavala et al. 1991). Confocal imaging demonstrated co-localization of TSPO and gp91^phox^ (Guilarte et al. 2016). These data showing a functional connection led Guilarte et al. (2016) to hypothesize that in microglia TSPO may transfer heme from mitochondria to the gp91^phox^ apoprotein in the ER by directly binding to it, possibly at the MAM, allowing mature gp91^phox^ to assemble with other subunits and form active NOX2. A follow-up paper used immunofluorescence microscopy, co-immunoprecipitation, and a proximity ligation assay to demonstrate a direct physical association of TSPO with gp91^phox^ and another NOX2 subunit p22^phox^ in murine microglia, and this interaction was reduced when microglia were activated by lipopolysaccharide treatment (Loth et al. 2020). However, TSPO’s connection to NOX in some mammalian cell lines appears to be indirect. Upregulation of TSPO increased the formation of a PKA-ACBD3 complex with TSPO and VDAC, leading PKA to phosphorylate VDAC, causing inhibition of Ca^2+^ uptake into mitochondria and an increase in cytosolic Ca^2+^, thereby activating the Ca^2+^-dependent isoform NOX5 and increasing ROS production (Gatliff et al. 2017). In a potentially similar indirect way, TSPO-dependent Ca^2+^ release from mouse retinal microglial mitochondria induced production of ROS by NOX1, which could be decreased by application of the TSPO ligand XBD173 (Wolf et al. 2020).

Plants possess many NADPH oxidase homologs (also called respiratory burst homologs or RBOHs) that are involved in many types of signaling and stress responses [reviewed in (Chapman et al. 2019)]. Extracellular ROS production in *Arabidopsis* in response to pathogen attack was reported to require the genes *RBOH*D and *RBOH*F, homologs to gp91^phox^ that encode components of a plasma membrane NADPH oxidase (Torres et al. 2002). Another report attributed this ROS production to a cell wall peroxidase (Bindschedler et al. 2006). Similar extracellular ROS production in response to fungal elicitor in the moss *P. patens* was shown to require *Pp*TSPO1 and was attributed to a cell wall peroxidase (Lehtonen et al. 2012). One commonality is that NADPH oxidases and peroxidases both require heme cofactors, potentially supplied by TSPO. However, no direct binding of either protein to TSPO has been reported in plants. Interestingly, ROS derived from NOX has been shown to regulate autophagy induced by nitrogen or carbon deficiency stress, but not constitutive autophagy, in wheat root tips (Jing et al. 2020). Although a connection to TSPO was not investigated in that report, plant TSPO has been associated with autophagy (see next section).

### Autophagy-Related proteins and selective autophagy receptors

TSPO has been associated with the process of autophagy as a means of combatting stress. Autophagy removes damaged or excess molecules or entire organelles by putting them into double-membraned vesicles (autophagosomes), which then carry the damaged cargo to the vacuole or lysosomes for degradation or to the cell membrane for secretion [for reviews, see (Michaeli and Galili 2014; Anding and Baehrecke 2017; Abdrakhmanov et al. 2020)]. The process involves recognition of the molecule or organelle to be removed by selective autophagy receptors (SARs), followed by movement into the autophagosome, and finally fusion of the autophagosome with the vacuole, lysosome, or cell membrane. SARs bind to the autophagosome via ubiquitin-like ATG8/LC3 proteins which, upon lipidation with phosphatidylethanolamine, bind to the forming autophagosomal membrane and thereby recruit SARs or other associated proteins (Anding and Baehrecke 2017). SARs interact with ATG8 through a consensus ATG8-interacting motif (AIM; also called LC3-interacting region or LIR).

The *Arabidopsis* TSPO was shown to be degraded by autophagy (Vanhee et al. 2011a). Autophagy may also provide a route for heme degradation in plants (Veljanovski and Batoko 2014), as heme binding was required for *At*TSPO autophagy (Hachez et al. 2014). *At*TSPO has an AIM motif (LYLYL, amino acids 121–125) (Vanhee et al. 2011a) (Fig. 1, green box) and co-purified with ATG8 in a manner requiring this motif (Hachez et al. 2014). The moss *Pp*TSPO also has 5 putative AIM motifs (Lehtonen et al. 2012), suggesting that binding of plant TSPO with ATG8 is unlikely to be exclusive to *Arabidopsis*, although it has been suggested that autophagy-dependent degradation of TSPO could be unique to plants (Jurkiewicz et al. 2018). Physical binding of animal TSPO to ATG8 or other autophagy-specific proteins has not been reported. One group has suggested that the TSPO-autophagy connection in mammals is indirect via ROS (Gatliff et al. 2014, 2017) and not due to direct binding of TSPO to ATG proteins. Both human and mouse TSPOs possess potential AIM motifs in a similar location as the AIM motif in *Arabidopsis*, suggesting a direct interaction could be possible. However, this motif is also present in *Rs*TSPO from a bacterium that does not have an autophagic pathway. In *R. sphaeroides* it might participate in a secretory pathway for porphyrins (Yeliseev and Kaplan 1999), analogous to yeast and mammalian secretory autophagy (Ponpuak et al. 2015; Keulers et al. 2016) which, when normal autophagy is defective or saturated, may be used for waste disposal by re-routing cargo to the extracellular environment. In fact, some bacterial pathogens produce proteins with AIM motifs that bind to host ATG proteins and thereby subvert clearance from the host cell via autophagy (McEwan 2017).

In *Arabidopsis*, *At*TSPO has also been shown to directly bind to and be a SAR for another protein (Fig. 3, bottom right). A search for potential *Arabidopsis* TSPO interacting partners found the plasma membrane intrinsic protein aquaporin PIP2;7 (Hachez et al. 2014). Pull-down assays and fluorescence imaging showed that *At*TSPO physically interacted with PIP2;7 in the ER and Golgi, prevented transport of PIP2;7 to the plasma membrane, and reduced PIP2;7-dependent water transport out of the cell (Hachez et al. 2014). When *Arabidopsis* seedlings co-expressing PIP2;7 and *At*TSPO were treated with autophagy inhibitors, the amounts of both proteins increased, while in an autophagy-deficient mutant, ABA increased the level of *At*TSPO but the level of PIP2;7 remained unchanged (Hachez et al. 2014). This suggests that both proteins are normally degraded by autophagy (Hachez et al. 2014). Binding of the signaling lipid phosphatidylinositol (4,5) bisphosphate (PI(4,5)P_2_) to the *At*TSPO N-terminal extension (via lysine/arginine pairs) was required for physical interaction with PIP2;7 and its subsequent degradation, although the N-terminus was not required for targeting of *At*TSPO alone to the vacuole for degradation (Jurkiewicz et al. 2018). PI(4,5)P_2_ has been reported to play a signaling or regulatory role in autophagosome formation and fusion with the lysosome (Tan et al. 2016; Baba et al. 2019) and autophagosomes can initiate near *At*TSPO-containing organelles (Veljanovski and Batoko 2014). A model was proposed in which the N-terminus of *At*TSPO contacts the autophagosome initiation membrane, which is enriched in PI(4,5)P_2_, and thereby targets the *At*TSPO-PIP2;7 complex to this site (Jurkiewicz et al. 2018). Because the N-terminal extension of the *At*TSPO is not well conserved in other plants, the generality of TSPO as a SAR for aquaporins is unknown.

In animals, TSPO is associated with diseases that are linked to defects in autophagy [reviewed in (Um and Yun 2017)]. TSPO has also been associated with MAMs, which are considered to play a role in autophagosome biogenesis (Vance 2014; Fan and Simmen 2019). In maturing cultured human erythroblasts, downregulation of TSPO or treatment with the TSPO ligand Ro5-4864 inhibited mitophagy (selective autophagy of mitochondria) and increased cell death (Moras et al. 2020). When TSPO levels are increased relative to VDAC in MEFs, ROS levels are increased and mitophagy is inhibited, possibly by affecting PARK2-mediated ubiquitination of mitochondria (Gatliff et al. 2014). In tanycytes (hypothalamic glial cells), addition of PK11195 or deletion of TSPO elicited AMP-activated protein kinase (AMPK)-dependent breakdown of lipid droplets (LD), increased the phosphorylation level of unc-51-like kinase 1 (a target of AMPK for autophagy initiation), induced autophagy-related genes and autophagy flux, and increased ATP production; the authors suggested that tanycytic TSPO affected energy balance through autophagy-regulated lipid metabolism (Kim et al. 2019) but did not report any direct binding of TSPO to any particular autophagy-related proteins. TSPO binds the acyl-coenzyme A binding protein (ACBP, also called DBI; see above), which has recently been shown to stimulate autophagy in mice, yeast, and *C.elegans* (Pedro et al. 2019; Charmpilas et al. 2020). Knockdown of TSPO has the same inhibitory effect on autophagy as the knockdown of ACBP (Bravo-San Pedro et al. 2019). ACBP is secreted by the yeast *Picia pastoris* and the fungus *Dictyostelium discoideum* in a manner requiring autophagy proteins (Duran et al. 2010; Manjithaya et al. 2010). The involvement of TSPO, perhaps as a SAR, was not investigated but would be consistent with these results.

### Other potential binding partners

The 800 kDa complex proposed to be involved in cholesterol import and containing the OMM proteins TSPO and VDAC also contains the IMM proteins CYP11A1, ATAD3A, and optic atrophy type 1 (OPA1) (Rone et al. 2012). CYP11A1 is the cytochrome P450 that cleaves the side chain of cholesterol to produce pregnenolone in the mitochondrial matrix (Slominski et al. 2015); pregnenolone is the common precursor of all steroid hormones (Papadopoulos 1993). ATAD3A is an ATPase located in the IMM at contact sites with the OMM, and is involved in regulation of steroid synthesis and mitochondrial morphology in a manner that depends on its ATP-bound state (Gilquin et al. 2010). OPA1 is an IMM protein involved in mitochondrial shape and fusion but not in hormone-induced steroidogenesis (Rone et al. 2012). No direct binding of TSPO to any of these proteins has been reported and TSPO’s connection to them is likely to be indirect, possibly via VDAC or 14-3-3 proteins.

Another potential partner, parkin (PARK2), the E3 ubiquitin ligase associated with Parkinson's disease, was proposed by Cho et al. to cooperate with the TSPO-VDAC complex to mediate responses against infection and wounding in *Drosophila,* since mutations in parkin + TSPO or parkin + VDAC showed similar effects as parkin homozygous mutants (Cho et al. 2015). Direct binding of these proteins was not addressed. Investigations of TSPO’s role in mitophagy indicate that the connection to parkin is indirect. TSPO overexpression in MEFs could prevent the necessary ubiquitination of mitochondria even though PARK2 was correctly recruited (Gatliff et al. 2014; Gatliff and Campanella 2015). The proposed model was that an increased ratio of TSPO to VDAC1 leads to an accumulation of ROS that counteracts the localized PARK2-dependent ubiquitination of mitochondria, thereby inhibiting mitophagy (Gatliff et al. 2014, 2017).

In the model plant *Arabidopsis*, expression of *At*TSPO was shown to stimulate phospholipase C (PLC) activity and PLC can promote responses to ABA and tolerance to hyperosmotic stress, situations also associated with plant TSPO (Jurkiewicz et al. 2020). However, there is currently no evidence of any direct binding of a phospholipase to TSPO.

In animals, peroxisome-proliferator-activated receptor alpha (PPARα) has been functionally linked to decreased TSPO expression by interfering with the ROS-activated NF-κB (nuclear factor kappa B), STAT (signal transducer and activator of transcription), and AP1 (activator protein 1) transcription factors that have binding sites in the TSPO gene promoter (Gatliff and Campanella 2016). PPARα and PPARγ have also been functionally associated with DBI, a known ligand of mammalian TSPO (Islinger et al. 2020). However, there are no reports of any direct binding of any PPARs to TSPO.

Use of a triple repeat of the C-terminal 14 amino acids of TSPO as a bait in a yeast two-hybrid screen found PRAX1, a ~ 200 kDa protein that was predominantly expressed in the brain and thymus but not in tissues typically rich in TSPO, such as steroidogenic tissues and liver. PRAX1 possesses several domains involved in protein–protein interaction, leading Galiegue et al. (1999) to suggest that PRAX1 may be an adaptor protein. However, in vitro binding assays could not confirm a direct interaction of TSPO and PRAX1 (Galiegue et al. 1999) and a later report showed that PRAX1 mRNA expression was mostly in neurons and not glia (Chardenot et al. 2002) where TSPO is usually expressed. Overall, these data suggest that binding between PRAX1 and TSPO may not be physiological.

Another potential TSPO interacting partner was proposed by Jurkiewicz et al. (2018): the NAC-domain-containing protein 89 (AtNAC089), a Golgi-processed bZIP-like transcription factor induced by osmotic and salinity stress that regulates genes involved in programmed cell death in plants. A large interactome project found that human TSPO could interact with a NAC089-related bZIP transcription factor CREB3L1 (cAMP Responsive Element Binding Protein 3- Like 1) that was listed as being involved in the unfolded protein response (UPR) [(Rolland et al. 2014) www.interactome-atlas.org]. This finding led the authors to suggest that TSPO could be directly or indirectly involved in regulation of the UPR (Rolland et al. 2014). CREB3L1 was also suggested to be involved in regulation of cargo proteins and Golgi expansion during upregulation of the secretory pathway in mammals (Garcia et al. 2017), which would fit with TSPO’s association with the Golgi (Guillaumot et al. 2009a) and autophagy (Hachez et al. 2014) in plants. The same large interactome project also suggested MEOX2, a mesodermal transcription factor that regulates vascular cell proliferation, and CLEC10A, a glycoreceptor reported to have a role in regulating adaptive and innate immune responses, as high-scoring potential binding partners of TSPO (Rolland et al. 2014), but no reports investigating the connection of TSPO to either of these proteins have been published.

A STRING database search for functionally-interacting partners for TSPO from *Pseudomonas* strains suggested several proteins for further study (Leneveu-Jenvrin et al. 2014). Not surprisingly, OprF, the *Pseudomonas* homolog of VDAC, was predicted to functionally interact with TSPO (Leneveu-Jenvrin et al. 2014). HemE is a uroporphyrinogen-III decarboxylase that produces coproporphyrinogen III, and this protein was also predicted to interact with *Rs*TSPO and *Sinorhizobium meliloti* TSPO (Leneveu-Jenvrin et al. 2014). Other predicted functional partners of TSPO were: GrxC, the monothiol glutaredoxin involved in bacterial oxidative stress response; PhrB, a photolyase also predicted to interact with *Rs*TSPO; the Kat B catalase, also predicted to interact with *S. meliloti* TSPO; hemolysin II/III, virulence factors that remove iron from heme; the adenine phosphoribosyltransferase Apt; a putative nucleoside-diphosphate-sugar epimerase Pfl01_0720 and the UDP-N-acetylmuramate-alanine ligase MurC, both involved in cell wall synthesis; orthologs of the thiol oxidoreductase PSPTO4367, involved in oxidative stress protection; and finally a putative hybrid histidine kinase Pfl01_2810, the interaction with which was suggested to be specific to *P. fluorescens* Pf0-1 (Leneveu-Jenvrin et al. 2014). No direct physical interaction of any of these proteins with any TSPO has been reported thus far. A functional association with the heat shock protein subunit GroL was also predicted (Leneveu-Jenvrin et al. 2014). GroEL is a chaperone for protein folding and has been co-purified as a contaminant with *Rs*TSPO (Li et al. 2013), suggesting a direct physical interaction but one involved in TSPO synthesis and/or refolding and not in TSPO function.

## Evolution of TSPO function and its protein binding partners

Amid its many proposed binding partners and functions, can an ancestral function and partner for TSPO be discerned? It seems reasonable to conclude that some part of TSPO function, along with aspects of its structure, must be conserved throughout evolution, as mammalian TSPO has been shown to functionally substitute for the bacterial *Rs*TSPO (Yeliseev et al. 1997) and mammalian TSPO can be altered to bind an aquaporin in a manner similar to the plant *At*TSPO (Jurkiewicz et al. 2020).

The most widely observed function in all organisms is the binding of porphyrins, with affinities ranging from low micromolar in bacteria (e.g., 5 µM in the cyanobacterium *F. diplosiphon* (Busch et al. 2017)) to low nanomolar in mammals (e.g., 15–40 nM in rat kidney mitochondria (Verma et al. 1987). Evidence of porphyrin binding is often associated with TSPO’s involvement in oxidative stress (Vanhee et al. 2011a; Batoko et al. 2015a, 2015b; Guo et al. 2015; Guilarte et al. 2016). However, TSPO’s role in mammalian porphyrin metabolism has been questioned (Banati et al. 2014; Zhao et al. 2016). Indeed, the TSPO knock-out mouse model was reported to have normal PPIX metabolism (Banati et al. 2014; Middleton et al. 2015). However, the need for TSPO was shown to relate to the mitochondrial energetic state in mammalian liver cells (Pastorino et al. 1994). Knock-out mice showed decreased mitochondrial respiration and ATP production (Banati et al. 2014), while knock-out *Drosophila* had reduced mitochondrial respiration and increased mitochondrial oxidative stress (Lin et al. 2014). These observations suggest that TSPO may not be involved in routine porphyrin synthesis, but possibly in regulating porphyrin levels under stress conditions and/or in relation to mitochondrial energy metabolism in mammals.

In higher plants, the role of porphyrins and oxidative stress are strongly connected (Frank et al. 2007; Guillaumot et al. 2009a; Vanhee et al. 2011a) [for a short review, see (Batoko et al. 2015b)]. Plants have been proposed to use TSPO and porphyrins to fine tune ROS homeostasis based on dual roles for porphyrins in ROS signaling: heme and PPIX may generate ROS themselves in the presence of light, but heme is also a cofactor of ROS scavengers and some products of heme breakdown (such as biliverdin) are antioxidants (Cui et al. 2016). Batoko et al. (2015a) proposed that, instead of acting as porphyrin transporters, TSPOs may bind and sequester heme and PPIX as a protection mechanism against ROS generation by free porphyrins, while some TSPOs may offer additional protection by light-induced degradation of PPIX (Ginter et al. 2013; Guo et al. 2015). On the other hand, Zeno et al. (2012) suggest that ROS generation modulated by TSPO could be a mechanism to prevent porphyrin accumulation. By regulating ROS and porphyrin levels, TSPO may curb prolonged or excessive expression of stress-related genes. This widespread binding of porphyrins in response to stress may indicate the most ancestral TSPO function (Batoko et al. 2015a) (Fig. 3, top). However, the key question of how porphyrin binding affects TSPO interactions with itself or other proteins remains to be addressed.

The handing of porphyrins to porin for export in bacteria, similar to what has been suggested for *R. sphaeroides* (Yeliseev and Kaplan 1995, 1999; Oh and Kaplan 2001), hints that porin may have been an ancestral binding partner for TSPO. This partnership may have later evolved into TSPO and VDAC moving porphyrins into or out of eukaryotic mitochondria or chloroplasts (Fig. 3, center). Mammalian TSPO may bind PPIX from the cytoplasm and transfer it to VDAC for import into the mitochondria for further processing into heme, making this process key to synthesis of many heme proteins needed for steroid synthesis and ROS production (Guilarte et al. 2016; Loth et al. 2020). A wealth of potential new partners and functions was opened up as TSPO gained the ability to bind to other proteins, particularly 14-3-3 proteins (Aghazadeh et al. 2012, 2014) and AKAPs (Liu et al. 2003; Fan et al. 2010; Desai et al. 2020) which act as scaffolds to bring other proteins together (Chevalier et al. 2009). A recently discovered partnership is TSPO’s association with PKA and AKAPs in the NAM, as a eukaryotic evolutionary adaptation to allow communication between the nuclear and mitochondrial genomes (Desai et al. 2020). However, porphyrin binding has been maintained while binding partners were changed or added. For example, although import of cholesterol into mammalian mitochondria involves a large complex of proteins in addition to TSPO (Liu et al. 2006; Rone et al. 2012)(Fig. 3, bottom left), TSPO’s ancestral porphyrin-binding ability is not lost upon strengthening of cholesterol binding by the addition of a LAF motif (Li et al. 2015a)(Fig. 1, blue box), and cholesterol import is still influenced by what may be an ancient partnership between TSPO and VDAC/porin.

## Summary

TSPO is an ancient protein whose varied and complex functions appear to be dictated by its location and its orientation in the membrane, as well as its binding partners. In many aspects of its behavior TSPO resembles a receptor or sensor; indeed, it shows some structural similarity to GPCRs (Li et al. 2016). In plants, however, there is good evidence for a heme translocator function in association with autophagy (Vanhee et al. 2011a; Hachez et al. 2014; Jurkiewicz et al. 2018). Nearly all TSPOs tested bind at least some porphyrins at high affinity and TSPO is implicated in porphyrin metabolism and transport (Verma et al. 1987; Verma and Snyder 1989; Taketani et al. 1995; Yeliseev and Kaplan 1999; Wendler et al. 2003; Papadopoulos et al. 2006; Frank et al. 2007; Guillaumot et al. 2009a; Vanhee et al. 2011a; Hachez et al. 2014; Batoko et al. 2015a, 2015b; Busch and Montgomery 2015b; Guo et al. 2015; Guilarte et al. 2016; Kobayashi et al. 2016; Busch et al. 2017). Although it may not be a direct porphyrin transporter on its own, in association with VDAC/porin it is linked to movement of porphyrins in or out of cells and into subcellular organelles (Yeliseev and Kaplan 1995; Wendler et al. 2003; Lindemann et al. 2004; Marginedas-Freixa et al. 2016). In mammals, TSPO evolution has led to an apparent regulatory role in the specialized function of cholesterol transport in steroidogenic tissues while maintaining the ancient partnership with VDAC/porin and adding on many more protein partners to form a large complex to accomplish and regulate this critical function (Liu et al. 2006; Rone et al. 2009, 2012). As TSPO gained the ability to bind 14-3-3 proteins (Aghazadeh et al. 2012, 2014) and ACBD proteins such as DBI/ACBP/ACBD1 (Guidotti et al. 1983; Papadopoulos et al. 1991, 1992; Papadopoulos 1993; Garnier et al. 1994a; Rone et al. 2009) or ACBD3/PAP7 (Li et al. 2001; Liu et al. 2003; Fan et al. 2010), evidence supports a gain of function from these new partnerships. TSPO, with its binding partners, exhibits significant involvement in many areas of metabolism and stress related activity. Researching TSPO’s interactions with newly-discovered and lesser-known binding partners will no doubt expand our understanding of this ancient regulatory protein which holds significant interest as a biomarker and participant in disease processes.

## Data Availability

Not applicable.

## Abbreviations

ABA: Abscisic acid
ACBD1: Acyl-coA binding domain-containing protein 1
ACBD3: Acyl-coA binding domain-containing protein 3
ACBP: Acyl-coA binding protein
AIM: Atg8-interacting motif
ANT: Adenine nucleotide transporter
*At*TSPO: TSP from *Arabidopsis thaliana*
CRAC: Cholesterol recognition/interaction amino acid consensus motif
DBI: Diazepam binding inhibitor
ER: Endoplasmic reticulum
IMM: Inner mitochondrial membrane
LD: Cytoplasmic neutral lipid droplets
MEFs: Mouse embryonic fibroblasts
MPTP: Mitochondrial permeability transition pore
NOX: NADPH oxidase
ODN: Octadecaneuropeptide product of DBI processing (amino acids 33–50)
OMM: Outer mitochondrial membrane
NAM: Nucleus-associated mitochondria
PAP7: Peripheral benzodiazepine receptor-associated protein 7
PBR: Peripheral benzodiazepine receptor
PI(4,5)P2: Phosphatidylinositol-(4,5)-bisphosphate
PKA: Cyclic AMP-dependent protein kinase
PKARIA: Protein kinase-A regulatory subunit Iα
PKC: Calcium-dependent protein kinase
PLC: Phospholipase C
PPIX: Protoporphyrin IX
ROS: Reactive oxygen species
*Rs*TSPO: TSPO from *Rhodobacter sphaeroides*
SAR: Selective autophagy receptor
StAR: Steroidogenic acute regulatory protein
TTN: Triakontatetraneuropetide product of DBI processing (amino acids 17–50)
TSPO: Tryptophan rich sensory protein
VDAC: Voltage-dependent anion channel
